# Pregnancy associated plasma protein-A links pregnancy and melanoma progression by promoting cellular migration and invasion

**DOI:** 10.18632/oncotarget.3643

**Published:** 2015-04-10

**Authors:** Prashanth Prithviraj, Matthew Anaka, Sonja J McKeown, Michael Permezel, Marzena Walkiewicz, Jonathan Cebon, Andreas Behren, Aparna Jayachandran

**Affiliations:** ^1^ Ludwig Institute for Cancer Research, Melbourne-Austin Branch, Cancer Immunobiology Laboratory, Heidelberg, VIC, Australia; ^2^ Olivia Newton-John Cancer Research Institute, Olivia Newton-John Cancer and Wellness Centre, Heidelberg, VIC, Australia; ^3^ Department of Medicine, University of Melbourne, Victoria, Australia; ^4^ School of Cancer Medicine, La Trobe University, Victoria, Australia; ^5^ Department of Anatomy and Neuroscience, University of Melbourne, Victoria, Australia; ^6^ Mercy Hospital for Women, Heidelberg, VIC, Australia

**Keywords:** PAPPA, melanoma, pregnancy, EMT, invasion

## Abstract

Melanoma is the most common cancer diagnosed in pregnant women and an aggressive course with poorer outcomes is commonly described during pregnancy or shortly after childbirth. The underlying mechanisms for this are not understood. Here, we report that melanoma migration, invasiveness and progression are promoted by pregnancy-associated plasma protein-A (PAPPA), a pregnancy-associated metalloproteinase produced by the placenta that increases the bioavailability of IGF1 by cleaving it from a circulating complex formed with IGFBP4. We show that PAPPA is widely expressed by metastatic melanoma tumors and is elevated in melanoma cells exhibiting mesenchymal, invasive and label-retaining phenotypes. Notably, inhibition of PAPPA significantly reduced invasion and migration of melanoma cells *in vitro* and *in vivo* within the embryonic chicken neural tube. PAPPA-enriched pregnancy serum treatment enhanced melanoma motility *in vitro*. Furthermore, we report that IGF1 can induce the phenotypic and functional effects of epithelial-to-mesenchymal transition (EMT) in melanoma cells. In this study, we establish a clear relationship between a pregnancy-associated protein PAPPA, melanoma and functional effects mediated through IGF1 that provides a plausible mechanism for accelerated melanoma progression during pregnancy. This opens the possibility of targeting the PAPPA/IGF1 axis therapeutically.

## INTRODUCTION

Cancer in pregnancy is on the rise [1]. Melanoma is the most common malignancy encountered during pregnancy and dismal outcomes are recognised to occur in patients who are pregnant at time of diagnosis [1–3]. An increased risk of recurrence of melanoma during pregnancy has been reported [3]. There are no standard guidelines of management in these patients and young women who have been previously treated for melanoma are advised to delay or avoid pregnancy to reduce the risk of recurrence.

While accumulating evidence supports a complex multi-step progression to metastasis, there is a great need to identify early metastasis-promoting events such as those that promote invasion. In melanoma, label-retention assays have been used to enrich a sub-population of slow-cycling cells which exhibit a greater capacity for invasiveness, efficient tumor-initiating capacity and have properties associated with stemness, epithelial-to-mesenchymal transition (EMT) and resistance to chemotherapy [4, 5]. We recently identified pregnancy-associated plasma protein-A (*PAPPA*) as a candidate gene with enriched expression in melanoma cells with a label-retaining phenotype [4].

PAPPA is a metalloproteinase that modulates Insulin-like growth factor (IGF) activity. It was initially found at high concentration in the plasma of pregnant women and subsequently has been implicated as a multifunctional modulator of a number of pathologic processes [6–9]. It is the principal physiological regulator of Insulin-like growth factor-binding protein (*IGFBP*)-4 and cleaves the IGFBP4/IGF1 complex, releasing IGF1 and thus modulating local IGF1 bioavailability [10]. Emerging evidence suggests that disrupting the regulation of IGF1 availability by aberrant expression of PAPPA can impact tumor biology [11, 12]. However, the role of PAPPA in melanoma progression has not previously been described.

Here we examine the functional role of PAPPA during melanoma progression and demonstrate that PAPPA activity modifies melanoma migration and invasiveness. This may explain the long-recognised, but poorly understood, link between melanoma progression and pregnancy.

## RESULTS

### PAPPA is highly and aberrantly expressed in human metastatic melanoma tumors and prognosticates clinical outcome

**Figure 1:**
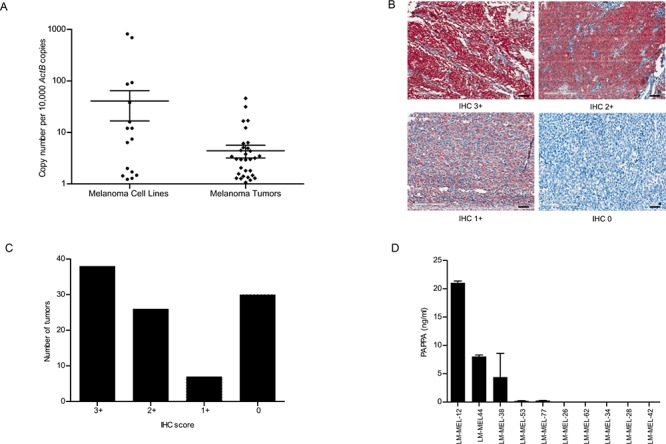
PAPPA expression in melanoma cell lines and tumors **A.** qRT-PCR analysis of *PAPPA* expression in a panel of metastatic melanoma cell lines and tumors compared to *ActB*. **B.** Representative images of PAPPA expression in malignant melanoma TMAs (scale bar = 100 μm). Positive PAPPA immunostaining was graded as IHC (3+, 2+ and 1+). **C.** Histogram shows number of tumors scored by IHC and PAPPA positivity in melanoma TMAs. **D.** Conditioned media from melanoma cell lines were collected and subjected to PAPPA ELISA.

We examined *PAPPA* mRNA expression by quantitative real-time RT-PCR (qRT-PCR) in a panel of human melanoma cell lines that were derived from resected melanoma metastases and found it to be variably expressed (Figure 1A). We also examined expression of *PAPPA* in 47 melanoma patient tumor samples and detected *PAPPA* expression in 87% of metastatic tumors tested (Figure 1A). PAPPA protein expression patterns in melanoma tumors were determined by immunohistochemical (IHC) staining of tissue microarrays (TMA) comprising of tumors from 103 patients with stage III and IV metastatic melanoma. IHC staining was graded in three categories – IHC 3+, 2+ and 1+. Cytoplasmic and membranous PAPPA expression was detected in 73% of metastatic melanoma patient tumors (Figure 1B & 1C).

We interrogated publicly available transcriptome profiling studies that have produced high-risk versus low-risk melanoma signatures. Two such datasets (Johnsson *et al*, and TCGA (http://bioinformatica.mty.itesm.mx:8080/Biomatec/SurvivaX.jsp) report expression clustering analysis that differentiate a total of 234 melanoma sample cohorts into high-risk and low-risk based on related transcriptome profiles [13–15]. *PAPPA* expression statistically significantly correlates with high-risk signature (*p* < 0.005), risk being reduced survival and risk of relapse (Supplementary Figure S1A&S1B).

As the secreted form of PAPPA has been implicated in the progression of some types of cancer, we next determined the secreted PAPPA levels in conditioned media by solid-phase ELISA in a subset of the high and low *PAPPA* expressing melanoma cell lines. ELISA detected significant amounts of PAPPA secretion in conditioned medium from cells lines with high *PAPPA* mRNA expression (Figure 1D). Immunohistochemical staining of cell line matched patient derived tumor biopsies revealed positive cytoplasmic and membranous staining for PAPPA (Supplementary Figure S2).

### Melanoma cells express major components of the IGF axis

**Figure 2:**
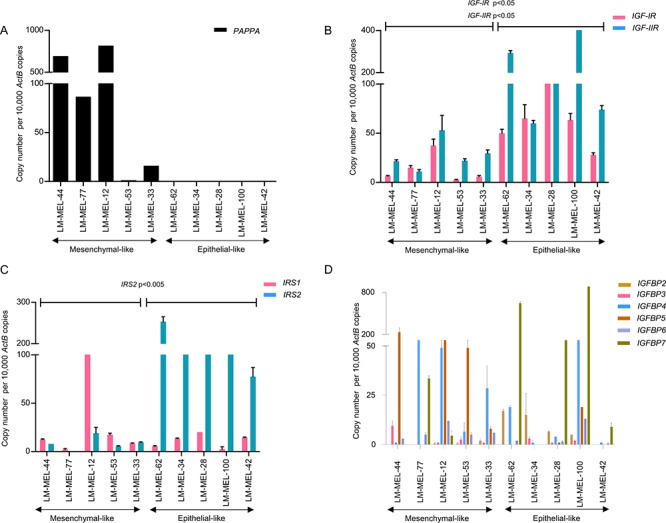
Expression of major components of IGF axis in melanoma cell lines **A.** *PAPPA* gene expression in mesenchymal-like and epithelial-like melanoma cell lines. Relative gene expression levels of **B.** *IGF* receptors (*IGF-IR* and *IGF-IIR*), **C.** Insulin Receptor Substrates (*IRS1* and *IRS2*) and **D.** *IGFBP*s (*IGFBP* 2–7) in mesenchymal-like and epithelial-like melanoma cell lines as analysed by qRT-PCR. Bars are mean values +/– SEM of three experiments in triplicate, Student's two-tailed *t*-test of the two sets (mesenchymal-like versus epithelial-like) are depicted as *p*-values.

The role of PAPPA in bio-modulation of IGF activity in malignancy has previously been reported [12, 16]. PAPPA exerts its biological effect through cleavage of IGFBP4 rendering IGF1 bioavailable at its receptor to enable local IGF action. The role of the IGF axis has been well documented in the induction of EMT in many cancers [17–19] often associated with a migratory and invasive phenotype [20, 21]. While not an epithelial cancer per se, EMT-like events have been described in melanoma [4, 22, 23]. We have previously categorised melanoma cell lines into those expressing E-cadherin as epithelial-like and those that lacked E-cadherin and only expressed N-cadherin as mesenchymal-like [4]. qRT-PCR revealed *PAPPA* mRNA expression only in mesenchymal-like melanoma cell lines and not those with an epithelial-like phenotype (Figure 2A).

We subsequently characterised the major components of the *IGF* axis in a panel of 54 melanoma cell lines by whole genome microarray expression profiling and report widespread and differential expression of major components of the *IGF* system (Supplementary Figure S3) [24]. We validated the microarray data using qRT-PCR in a subset of melanoma cell lines and data from both these techniques were well correlated. We noted higher expression of IGF-receptors, *IGF-IR* and *IGF-IIR* and IGF receptor substrates, *IRS1* and *IRS2* in the epithelial-like melanoma cell lines examined (Figure 2B & 2C). IGFBPs, the putative targets of proteolytic action of PAPPA were differentially expressed between mesenchymal-like and epithelial-like melanoma cells. *IGFBP4–6* were upregulated in mesenchymal-like melanoma cell lines when compared to epithelial-like cells. Notably, *IGFBP-4*, the primary substrate of PAPPA was upregulated in mesenchymal-like melanoma cells. *IGFBP-2*, *IGFBP-3* and *IGFBP-7* expression was elevated in epithelial-like melanoma cells (Figure 2D). This demonstrates the association between the PAPPA/IGF axis and a mesenchymal phenotype in melanoma.

### *In vitro and in vivo* silencing of *PAPPA* inhibits motility of melanoma cells

To evaluate the previously unknown role of PAPPA in melanoma, we utilised an embryonic chicken transplantation model that is gaining traction for melanoma tumor invasion studies *in vivo* [4, 25, 26]. This model is a useful tool for analysing cellular plasticity and invasion in an environment that is accessible surgically and that enables visualisation as well as the ability to manipulate migratory pathways [27, 28]. It involves the injection of melanoma cells into the neural tube of developing chick embryos, where cells acquire mesenchymal characteristics, become more motile and follow the migratory path of neural crest cells into more peripheral tissues [28].

**Figure 3:**
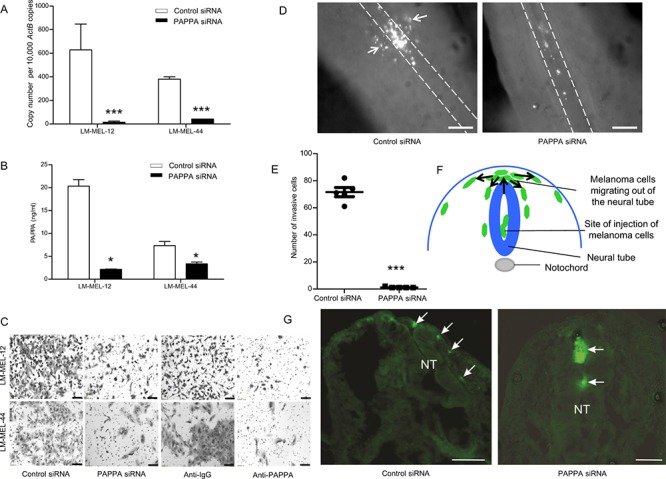
Silencing of PAPPA blocks motility of melanoma cells *in vitro* and *in vivo* Melanoma cells were plated and transfected with either control siRNA or PAPPA specific siRNA. After 72 hours, RNA was extracted and PAPPA **A.** qRT-PCR and **B.** ELISA was performed with conditioned medium. **C.** Melanoma cells were transfected with siRNAs as described, representative images of invasion as assessed by Boyden chamber transwell invasion assay were captured in LM-MEL-12 and LM-MEL-44 (scale bar = 100 μm). **C.** Invasive ability of melanoma cells after treatment with control anti-IgG or anti-PAPPA antibody were tested, images captured (scale bar = 100 μm). Melanoma cells were stained with CM-DiO, transfected with the control siRNA and PAPPA siRNA constructs and cultured as hanging drops to encourage aggregate formation. Similar sized aggregates were introduced into the neural tube of developing chicken and re-incubated within the egg for 2 days. **D.** Embryos were harvested and fluorescence pictures from whole-mounts taken (scale bar = 50 μm). White broken line shows the outline of the neural tube and the white arrows indicate melanoma cells that migrated out of the neural tube and into the surrounding tissue. **E.** Analysis of cell numbers infiltrating the surrounding tissue from several independent experiments (*n* = 5 for control siRNA and PAPPA siRNA) **F.** Schematic of a cross-section through the trunk region of the neural tube showing migration of melanoma cells. Melanoma cells are injected into the lumen of the neural tube and some remain in this position without migrating. Melanoma cells migrate into the region above the neural tube and they then migrate into surrounding tissue following pathways shown by arrows. **G.** Images from cross-sections of embryos. White arrows indicate melanoma cells and NT denotes neural tube, dorsal is to the top (scale bar = 100 μm). Values are mean +/– SEM of three independent experiments in triplicate (*p < 0.05, ***p < 0.0005).

We suppressed expression and secretion of PAPPA with two different siRNAs in two melanoma cell lines expressing high PAPPA levels, LM-MEL-12 and LM-MEL-44. Effective silencing of PAPPA was confirmed by qRT-PCR and ELISA (Figure 3A & 3B). The silencing of *PAPPA* in both cell lines was associated with significantly reduced cell invasion and antibody treatment with anti-PAPPA antibody decreased the invasive ability of melanoma cells (Figure 3C, Supplementary Figure S4) similarly.

Next, melanoma cells transfected with *PAPPA* siRNA or a non-targeting control were cultured as hanging drop for 24 hours and then introduced into the trunk neural tube of a developing chick embryo. *PAPPA* siRNA treated cells demonstrated a significant reduction in emigration from the neural tube *in vivo* into the surrounding tissue (Figure 3D & 3E). Cross-sections of chick embryo confirmed that numerous control siRNA treated cells migrate out of the neural tube in contrast to *PAPPA* siRNA treated cells that predominantly remain at the site of injection (Figure 3F & 3G). However, differences between control siRNA and *PAPPA* siRNA *in vivo* may have been exacerbated by the decreased proliferation seen in *PAPPA* siRNA treated cells after 72 hours. Overall, these results imply a prominent role for *PAPPA* in melanoma invasion *in vitro* and *in vivo*.

### Functional validation of *PAPPA* as a pro-migration gene in melanoma

**Figure 4:**
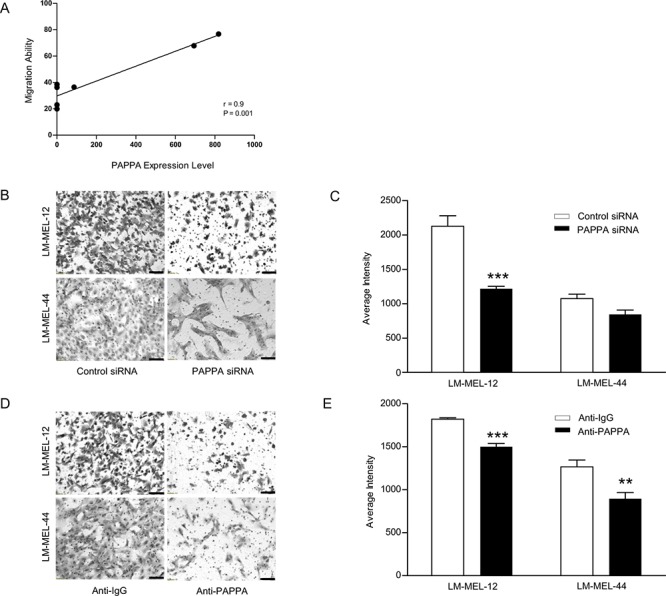
Ability of migration in melanoma positively correlates with PAPPA expression **A.** Migratory ability of melanoma cell lines was determined by transwell migration assay. The expression and secretion of PAPPA positively correlates with migration ability of melanoma cell lines. These data represent means +/– SEM performed in triplicates. The significance (*p* value) and correlation co-efficient (r) were calculated by Pearson's correlation analysis. **B.** Melanoma cells were transfected as described, representative images of migration in LM-MEL-12 and LM-MEL-44 are shown (scale bar = 100 μm). **C.** Graphs show average intensities of migrated cells after crystal violet staining calculated in K counts mm^2^ using Odyssey Software. **D.** Migratory ability of melanoma cells after treatment with control anti-IgG or anti-PAPPA antibody were tested, images captured (scale bar = 100 μm) and **E.** quantified as above. Values are mean +/– SEM of four independent experiments in triplicate (**p < 0.005, ***p < 0.0005).

We next sought to investigate whether reduced PAPPA expression would lead to a reduction in melanoma cell migration and we found that high *PAPPA*-expressing cell lines exhibited statistically significant positive correlation with migration when compared to low *PAPPA* expressing lines (Figure 4A). siRNA silencing of *PAPPA* in two cell lines was associated with significantly reduced cell migration in transwell assays (Figure 4B & 4C). Melanoma cells co-incubated with an anti-PAPPA antibody showed significant decrease in the migratory ability (Figure 4D & 4E). To further delineate the role of *PAPPA* in migration, wound healing assays were performed. Cells transfected with *PAPPA* siRNA showed significant inhibition of migration with delayed closure of wound when compared to control siRNA transfected cells after 24 hours (Supplementary Figure S5).

We measured proliferation of cells treated with siRNA against *PAPPA* and controls to confirm that effects on migration and invasion were not secondary to changes in cell viability during the assay. No difference in proliferation was observed at 24 hours, which was the time point after siRNA treatment measured in the migration and invasion assays (Supplementary Figure S6). However, inhibition of cell growth was observed 72 hours after silencing of *PAPPA*.

### Migratory capability of melanoma cells is enhanced by co-culture with PAPPA-rich human pregnant serum

**Figure 5:**
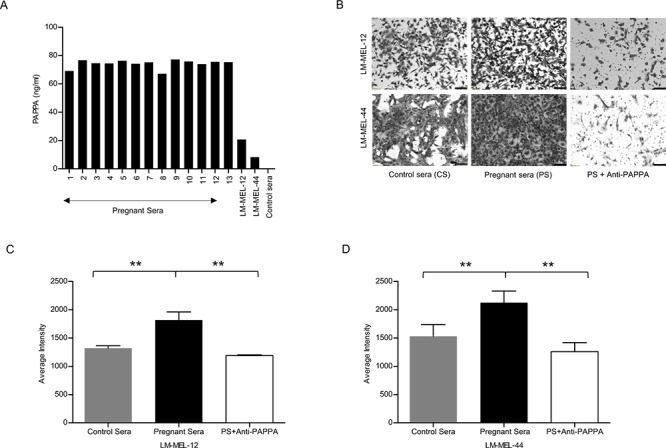
Pregnant serum enhances migratory capability of melanoma cell lines *in vitro* **A.** PAPPA ELISA was performed on sera collected from healthy pregnant women, sera from non-pregnant control and conditioned media from LM-MEL-12 and LM-MEL-44. **B.** Melanoma cell lines LM-MEL-12 and LM-MEL-44 were incubated with the different sera or a combination of PS and anti-PAPPA antibody for 48 hours and were subjected to transwell migration assays. Representative images of migration are as shown after 24 hours (scale bar = 100 μm). The graphs show average intensities of migrated **C.** LM-MEL-12 and **D.** LM-MEL-44 cells as measured after crystal violet staining. Values are mean +/– SEM of three independent experiments in triplicate (**p < 0.005).

PAPPA is produced in high levels by synctiotrophoblast cells in human placenta and is enriched in pregnant sera [8, 29]. To determine whether elevated PAPPA in maternal circulation during pregnancy can accelerate melanoma progression, we initially examined the level of secreted PAPPA in pregnant sera. As expected, higher amount of PAPPA was detected by ELISA in thirteen from pregnant women (PS) sera collected in third trimester of gestation compared with non-pregnant control serum and melanoma cell lines (Figure 5A). Co-culture of melanoma cells with human PS for 48 hours enhanced *in vitro* migration in comparison to control sera (Figure 5B, 5C & 5D). To test the specific effect of PAPPA within these sera in mediating migration, we assessed migration after addition of a PAPPA neutralising antibody. Significant abrogation of pregnancy serum-mediated migration with neutralising anti-PAPPA antibody was detected (Figure 5B, 5C & 5D). These results show that PAPPA protein in sera of pregant women causes melanoma cell migration.

In LM-MEL-62, an epithelial-like melanoma cell line with no inherent PAPPA production, co-culture with PS lead to change in morphology with induction of cell spreading and invasive protrusions as compared to control sera. This was associated with decrease in differentiation markers MelanA (*MLANA*), Tyrosinase (*TYR)* and *GP100* (data not shown).

### IGF1 is potent elicitor of EMT in melanoma

We next examined genomic changes in the PAPPA locus in a panel of different tumor types from The Cancer Genome Atlas (TCGA) (http://www.cbioportal.org) [13, 14] and noted a comparatively high occurrence of PAPPA aberrations across a panel of 262 sequenced melanoma tumors (Supplementary Figure S7). A significant correlation between *PAPPA* and *IGF1* expression was observed (Odds ratio = 3.7,  *p* = 0.031, Fisher's exact test). Additionally, *PAPPA* was significantly co-expressed with *CDH*2 (*N-cadherin*), a recognised mesenchymal marker.

**Figure 6:**
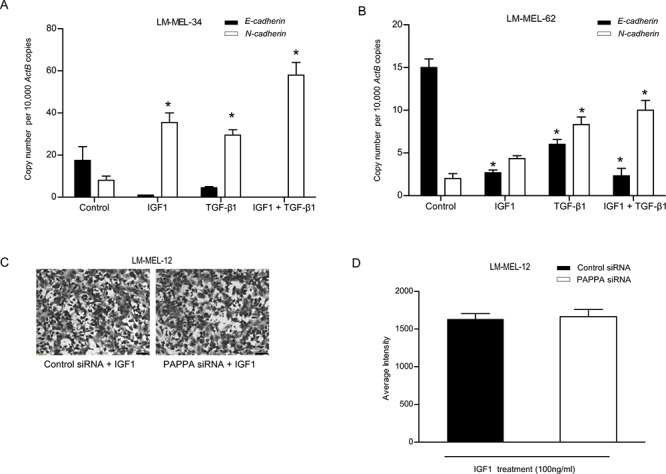
IGF1 induces Epithelial-to-Mesenchymal Transition (EMT) in human melanoma Epithelial-like melanoma cells **A.** LM-MEL-34 and **B.** LM-MEL-62 were plated and incubated with or without IGF1, TGF-β1 or a combination of both IGF1 and TGF-β1 for 3 days at indicated doses. qRT–PCR analysis of EMT markers E- and N-cadherin expression was assessed. **C.** LM-MEL-12 was plated and treated with control or PAPPA siRNA with or without IGF1 at 100 ng/ml and transwell migration assay was performed. Representative images of migration as shown after 24 hours (scale bar = 100 μm) and **D.** migratory ability of melanoma cells after treatment with control or PAPPA siRNA with IGF1 was quantified as average intensity of transwell membrane. The data are representative of three independent assays carried out in triplicate (mean +/–SEM, *n* = 3, *p < 0.05).

Previous studies have shown that IGF1 can elicit EMT in cancers [17, 19, 30], so we assessed the ability of IGF1 to induce an EMT in two epithelial-like melanoma cell lines LM-MEL-34 and LM-MEL-62, respectively. In both, IGF1 induced EMT by repressing E-cadherin and concomitantly enhancing N-cadherin expression (Figure 6A & 6B). We and others have previously induced EMT in melanoma cells with Transforming growth factor (TGF)-β1 [4, 31, 32]. Interestingly, we found that IGF1 was a more potent inducer of EMT in comparison to TGF-β1 in LM-MEL-34 (Figure 6A). A combination of both TGF-β1 and IGF1 showed an additive effect in the induction of EMT in both lines (Figure 6A & 6B). As PAPPA is a known modulator of IGF bioavailability, we tested whether bioactive, non IGFBP-bound IGF1 could restore the migratory ability of melanoma cells after silencing *PAPPA* gene expression. Figure 6C & 6D confirms restoration of migration ability after PAPPA knockdown with addition of IGF1. Taken together, these findings indicate that *PAPPA* can enhance melanoma cell migration by inducing IGF-mediated EMT.

## DISCUSSION

We have previously reported our studies of cellular heterogeneity in melanoma and the functional characteristics of cellular subpopulations, including slow-proliferating ‘label-retaining’ cells with invasive mesenchymal characteristics as well as non-invasive ‘epithelial’-like cells [4]. We found differential expression of PAPPA and have now extended those investigations to show that *PAPPA* expression was associated with mesenchymal-like but not epithelial-like melanoma cell lines. We demonstrate a role for PAPPA in cellular migration, invasion and proliferation. Inhibition with a neutralizing antibody against protein or siRNA, reduced the migration and invasion of melanoma cells both *in vitro* and *in vivo*. Conversely, exposure to PAPPA rich sera derived from pregnant women enhanced the migratory and invasive behaviour of these cells. Antibody-based specific blockade of PAPPA in this context confirmed a key role of the protein in this effect. Taken together, these data support a role for PAPPA in melanoma progression, particularly in association with high levels of PAPPA protein in pregnancy.

Melanoma is the most common cancer diagnosed in women of child-bearing age and higher rates during pregnancy contribute to it being the commonest cancer of pregnant women [1]. In this setting, a more aggressive clinical course and poorer outcomes are recognised to occur [2, 33–35]. PAPPA is produced abundantly by placental syncytiotrophoblasts and secreted into the maternal circulation where its concentrations increase exponentially until term [8]. Further, PAPPA has long been recognised as a useful biomarker of fetal genetic disorders and adverse pregnancy outcomes [7, 36]. Here, we clearly demonstrate that sera derived from pregnant women increases melanoma cell migration, an effect which is effectively attenuated by neutralising PAPPA. This points to a probable role for PAPPA in accelerating the progression of melanoma that is observed in pregnancy.

A role for PAPPA promoting cancer is supported by several lines of evidence; PAPPA null mice display a reduced incidence of spontaneous tumor formation as compared to age matched controls [37], high levels of PAPPA have been associated with progression in other tumor types [11, 12, 16, 37, 38] and Pan *et al* demonstrated that secreted, rather than cell-associated PAPPA, was associated with cancer growth and progression [39]. We found that PAPPA is highly expressed and secreted by melanoma cells with a mesenchymal phenotype and our data suggests a role in melanoma promotion.

Our demonstration that *PAPPA* expression was associated with increased melanoma migration and invasion aligns with a recent study by Huang *et al* reporting a pro-migratory role of PAPPA in malignant pleural mesothelioma [12]. These findings were confirmed *in vivo* since *PAPPA* knockdown abrogated the invasive and plastic potential of melanoma cells within the developing chicken neural tube. We and others have previously utilised the chicken transplantation model to investigate the role of candidate genes in invasion of melanoma cells *in vivo* by perturbing gene expression [4, 26, 28, 40]. Interestingly, PAPPA expression has been detected in developing neural crest cells and since melanocytes are embryologically derived from neural crest, it is likely that the environmental signals in this model are pertinent to melanoma cell motility [25, 41].

PAPPA can act as an autocrine or paracrine regulator of IGF action and the proposed mechanism for enhancing cancer migration is by increasing IGF release following the proteolysis of IGFBP4 [10, 42]. IGF signalling is a central player in the induction and maintenance of EMT in several cancers including breast, hepatocellular cancer, thyroid and prostate cancer [17, 18, 43, 44]. Our finding that IGF1 elicits EMT in melanoma cells is consistent with these studies. Although we demonstrated induction of a mesenchymal phenotype by IGF1 and enrichment of PAPPA in mesenchymal-like melanoma cells, we did not exhaustively study the role of PAPPA in modulating alternative signalling pathways such as TGF-β, another potent inducer of EMT [45]. This becomes increasingly relevant as TGF-β has been reported to stimulate PAPPA production in multiple cancers, and also has been reported to tightly regulate IGFBP4 protease activity in various models [46]. We also report enhanced induction of EMT when TGF-β1 and IGF1 were combined, suggesting cooperation between TGF-β and IGF signalling to drive EMT during melanoma growth and metastasis. Additionally, melanoma cells express different components of the IGF system and the presence of more than one IGFBP in melanoma is suggestive of functional co-operation. Although PAPPA is the primary physiological IGFBP4 protease, it has also been reported to cleave IGFBP2, IGFPB3 and IGFPB5 [10]. Based on the multi-domain structure of PAPPA, it is conceivable that there may be other non-proteolytic mechanisms involved in promoting the various functions of PAPPA in melanoma.

Clinical trials targeting IGF signalling in cancer with IGF-IR receptor inhibitors alone or in combination have not generally been effective [47, 48]. Treatment failure has largely been attributed to the high degree of homology shared between IGF-IR and insulin receptors and interference with insulin activity causing severe hypoglycemia [48]. PAPPA inhibition has the potential of being able to target the IGF axis more selectively, and this indirect approach of targeting IGF receptor signalling has the advantages of fewer side effects and increased specificity [49].

In conclusion, our study provides strong evidence for an important role of PAPPA in melanoma progression. Indirect and specific inhibition of IGF-IR signalling by inhibition of PAPPA, alone or in combination with current treatments represents a promising approach which merits further evaluation as a new therapeutic target for melanoma. Moreover, the fact that PAPPA functionally drives invasion and migration of melanoma in addition to its physiological role of bio-modulation of IGF activity in pregnancy strongly suggests a biological mechanism involved in melanoma progression during pregnancy, further study of which may present individual gene targets suitable for therapeutic intervention, during pregnancy or just after childbirth.

## MATERIALS AND METHODS

### Cell culture and melanoma patient samples

Melanoma cell lines were established from resected melanoma metastases by mechanical dissociation of tissue with subsequent overnight digestion in media containing collagenase IV at 37°C. Established cell lines were Mycoplasma-tested using the MycoAlert test (Lonza Rockland, Inc., USA). All tissue donors provided written informed consent for tissue collection and research, which was covered by protocols approved by the Austin Health Human Research Ethics Committee, Melbourne, Australia (approval number H2012/04446). All cell lines were matched with their donors by HLA-typing and STR-profiling. Cells were cultured in RPMI1640 supplemented with 10% fetal calf serum (FCS) as described previously [24].

### qRT-PCR

RNA was extracted using the RNEasy kit (Qiagen, Germany). Reverse transcription was carried out using the High Capacity cDNA RT kit (Applied Biosystems, Life Technologies, USA). Following reverse transcription, qRT-PCR was performed using SYBR Green (Qiagen, Germany). Following primers were used: Beta-Actin (*ActB*) was used as internal control. *ActB* (forward) 5′-ctg gaa cgg tga agg tga ca-3′ and (reverse) 5′-cgg cca cat tgt gaa ctt tg-3′, *PAPPA* (forward) 5′-aac ccc aca cgg gta gag a-3′ and (reverse) 5′-aga gca ggg tga gga tac ca-3′, *E-cadherin* (forward) 5′-gcc gag agc tac acg ttc a-3′ and (reverse) 5′-gac cgg tgc aat ctt caa a-3′, *N-cadherin* (forward) 5′-ctc cat gtg ccg gat agc-3′ and (reverse) 5′-cga ttt cac cag aag cct cta c-3′, *IGFBP-1* (forward) 5′-cca tgt cac caa cat caa aaa-3′ and (reverse) 5′-cct tgg cta aac tct cta cga ctc-3′, *IGFBP-2* (forward) 5′-ggt ggc aag cat cac ctt-3′ and (reverse) 5′-acc tgg tcc agt tcc tgt tg-3′, *IGFBP-3* (forward) 5′-aac gct agt gcc gtc agc-3′ and (reverse) 5′-ggt ctt cct ccg act cac-3′, *IGFBP-4* (forward) 5′-cct cta cat cat ccc cat cc-3′ and (reverse) 5′-ggt cca cac acc agc act t-3′, *IGFBP-5* (forward) 5′-cta ccg cga gca agt caa g-3′ and (reverse) 5′-gtc tcc tcg gcc atc tca-3′, *IGFBP-6* (forward) 5′-tga cca tcg agg ctt cta cc-3′ and (reverse) 5′-cat ccg atc cac aca cca-3′, *IGFBP-7* (forward) 5′-act ggc tgg gtg ctg gta-3′ and (reverse) 5′-tgg atg cat ggc act cat a-3′, *IGF-IR* (forward) 5′-aaa aac ctt cgc ctc atc ct-3′ and (reverse) 5′-tgg ttg tcg agg acg tag aa-3′, *IGF-IIR* (forward) 5′-gcc tgt gtt cct tct cca g-3′ and (reverse) 5′-agg cca gtc agg tcg tac tc-3′, *IRS1* (forward) 5′-tat gcc agc atc agt ttc ca-3′ and (reverse) 5′-ttg ctg agg tca ttt agg tct t-3′, *IRS2* (forward) 5′-ttc ttg tcc cac cac ttg aa-3′ and (reverse) 5′-ctg aca tgt gac atc ctg gtg-3′.

### Immunohistochemistry and pathological evaluation

Paraffin embedded tissue slides were deparaffinised and rehydrated, endogenous peroxidise activity was blocked with 3% Hydrogen peroxide, antigen retrieval was performed in 10mmol/L citrate buffer, and nonspecific binding was blocked with blocking reagent. PAPPA antibody (Sigma Aldrich; HPA 001667) was applied at 1.5 μg/mL concentration and incubated overnight at 4°C, followed by 60 minute incubation with secondary anti-rabbit antibody HRP (Dako). The chromogen used was 3-amino-9-ethylcarbazole (AEC). Human placenta was used as the positive control for PAPPA. A negative control, for which the primary antibody was substituted with the same concentration of Rabbit IgG, was prepared simultaneously. Slides were scanned using a ScanScope XT (Aperio) and immunohistochemical reactivity was evaluated by two independent investigators. The expression of PAPPA was categorized into four grades: IHC 3+, strong staining; IHC 2+, moderate staining; IHC 1+, weak staining and IHC 0, no staining.

### PAPPA ELISA

PAPPA protein content in cell supernatant and human sera was measured by Quantikine human PAPPA ELISA kit (R&D Systems, USA) as per the manufacturer's instructions.

### Gene expression microarray analysis

Illumina HT-12 gene expression microarray data from the LM-MEL cell line panel were pre-processed as described previously [24]. Supervised hierarchical clustering of IGF axis genes was performed in Partek Genomics Suite using Pearson dissimilarity and average linkage.

### *PAPPA* knockdown

For transient siRNA transfection, cells at 30% confluence were transfected using a control siRNA and two different Silencer select siRNAs targeting PAPPA (s10040 & 104027) at 20nM final concentration (Ambion, USA) with Lipofectamine RNAiMAX according to the manufacturer's protocol (Invitrogen, USA). Cells were incubated with siRNA complex for 48 hours and then functional assays were performed.

### Invasion and migration assays

Invasion assays were performed in Boyden chamber inserts with Matrigel coating (Becton, Dickinson and Company, USA). Insert membranes were stained with 0.1% crystal violet solution (Sigma, USA) analysed by Cell Sensi Software. Cells were photographed with a monochromatic Olympus camera. Analysis of intensity of transwell membrane calculated in K counts mm^2^ with Odyssey LI-COR Scanner System (LI-COR Biosciences, USA) was utilised after crystal violet staining as a measure of migration and invasion abilities.

### Wound healing assay

After melanoma cells were seeded in a 6 well plate and allowed to reach confluency, a scratch or wound was made using a sterile pipette tip. Photographs of migrating cells were taken at the indicated time points and wound field surface area measurement was determined using Olympus software. Assays were performed in triplicate for each condition.

### *In vivo* avian neural crest model

The chicken embryo transplantation was performed as previously described [4]. Briefly, melanoma cells were treated with PAPPA-specific siRNAs or scrambled control siRNA as described and labelled with CM-DiO as per manufacturer's instructions (Invitrogen, USA). Cells were grown overnight in a hanging-drop fashion to allow the formation of aggregates. Fertile chicken eggs were incubated at 38°C for 48 hours prior to transplantation. Cell aggregates were harvested and carefully injected with a glass pipette into the trunk neural tube lumen of developing chicken embryos. The eggs were then sealed with adhesive tape and re-incubated for 2 days. After incubation, embryos were removed from the eggs and fixed with 4% paraformaldehyde and whole mounts or cross sections were analyzed for the localization of melanoma cells using a Lumar V12 Zeiss microscope.

### Human serum treatment

Melanoma cell lines LM-MEL-12 & LM-MEL-44 were incubated with normal human third trimester pregnant sera (1 in 5 dilution with RPMI1640) and normal human control sera (1 in 5 dilution with RPMI1640) for 48 hours at 37°C (Ethics approved by Mercy Health HREC-R13/13). Functional analysis of invasion and migration were performed after 48 hours treatment.

### IGF1 and TGF-β1 treatment

Melanoma cell lines LM-MEL-62 and LM-MEL-34 were treated with 10ng/ml IGF1 & 5ng/ml TGF-β1 (Pepro Tech Inc., USA). RNA was extracted 72 hours following treatment. For IGF1 rescue of migration experiments LM-MEL-12 was treated with 100ng/ml IGF1 for the indicated times.

### Proliferation assay

10, 000 cells per well were plated out in 96 well plates and treated as indicated. Relative cell numbers were measured using the CellTiter 96^®^ AQueous One Solution Cell Proliferation Assay (Promega Corporation, USA).

### Statistical analysis

All statistical comparisons of data sets were performed using Student's two-tailed *t*-test or ANOVA analysis in Prism software version 5.00 (GraphPad Software Inc).

## SUPPLEMENTARY FIGURES


