# Supplementary material for: Pregnancy associated plasma protein-A links pregnancy and melanoma progression by promoting cellular migration and invasion
Source: Oncotarget. 2015 Apr 10;6(18):15953–65. doi: 10.18632/oncotarget.3643 (PMC4599249; doi:10.18632/oncotarget.3643)
Supplement: Supplementary file 1 [file oncotarget-06-15953-s001.pdf]

SUPPLEMENTARY FIGURES

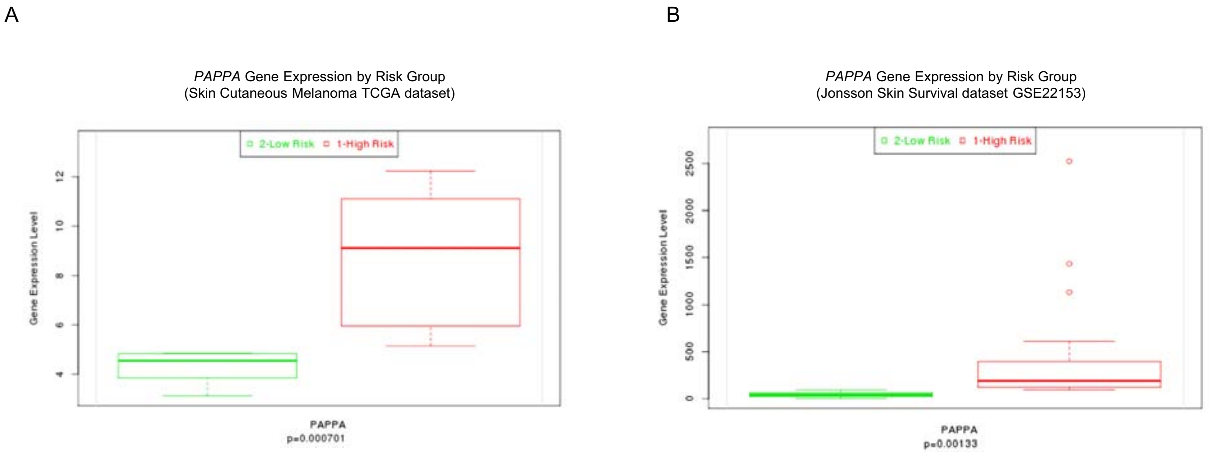

Supplementary Figure S1: PAPPA expression levels prognosticates clinical outcome in melanoma patients. *PAPPA* expression statistically significantly correlates with high-risk prognostic score ($p < 0.005$), risk being survival and risk of relapse in melanoma patients from publicly available datasets **A.** TCGA and **B.** Johnsson *et al* Skin Survival Dataset GSE22153.

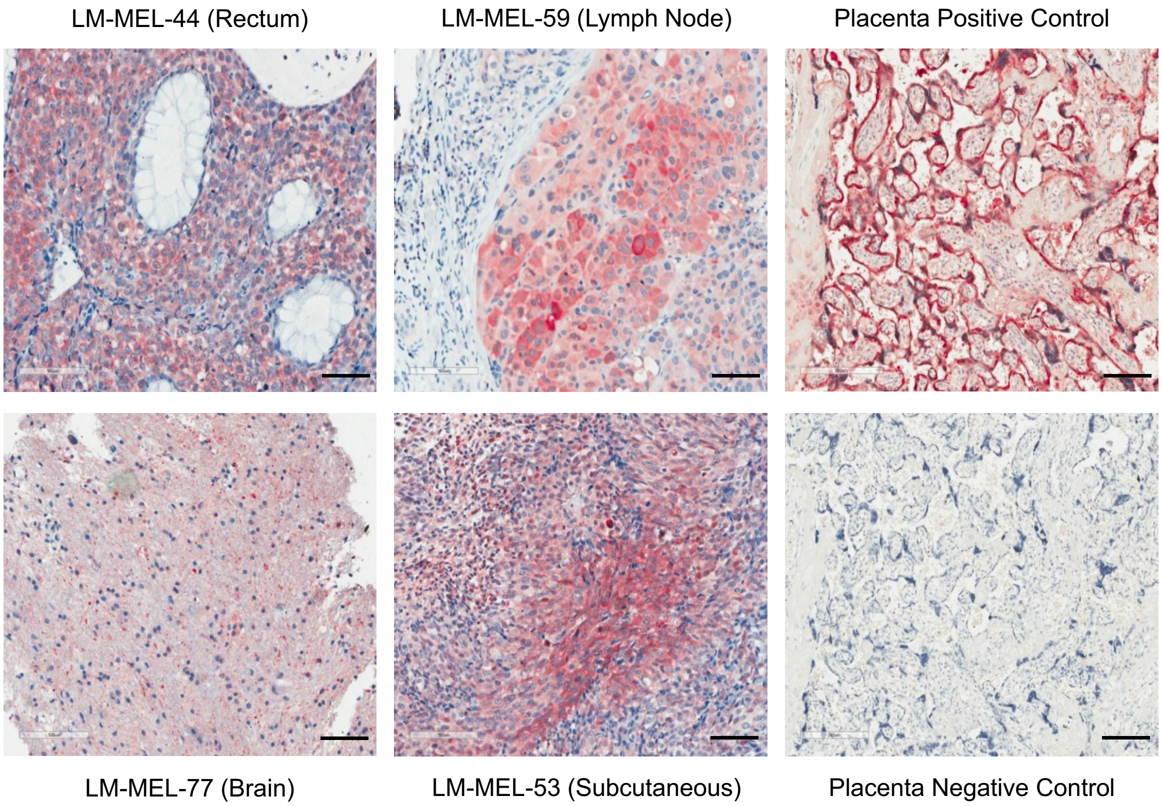

Supplementary Figure S2: Localisation of PAPPA in cell lines matched patient derived melanoma tumor biopsies. Immunostaining of PAPPA in melanoma tumors matched with melanoma cell lines LM-MEL-44, LM-MEL-59, LM-MEL-77 and LM-MEL-53. Human placenta was used as positive control for PAPPA. A negative control, for which the primary antibody was substituted with the same concentration of Rabbit IgG, was prepared simultaneously (scale bar = 100 µm).

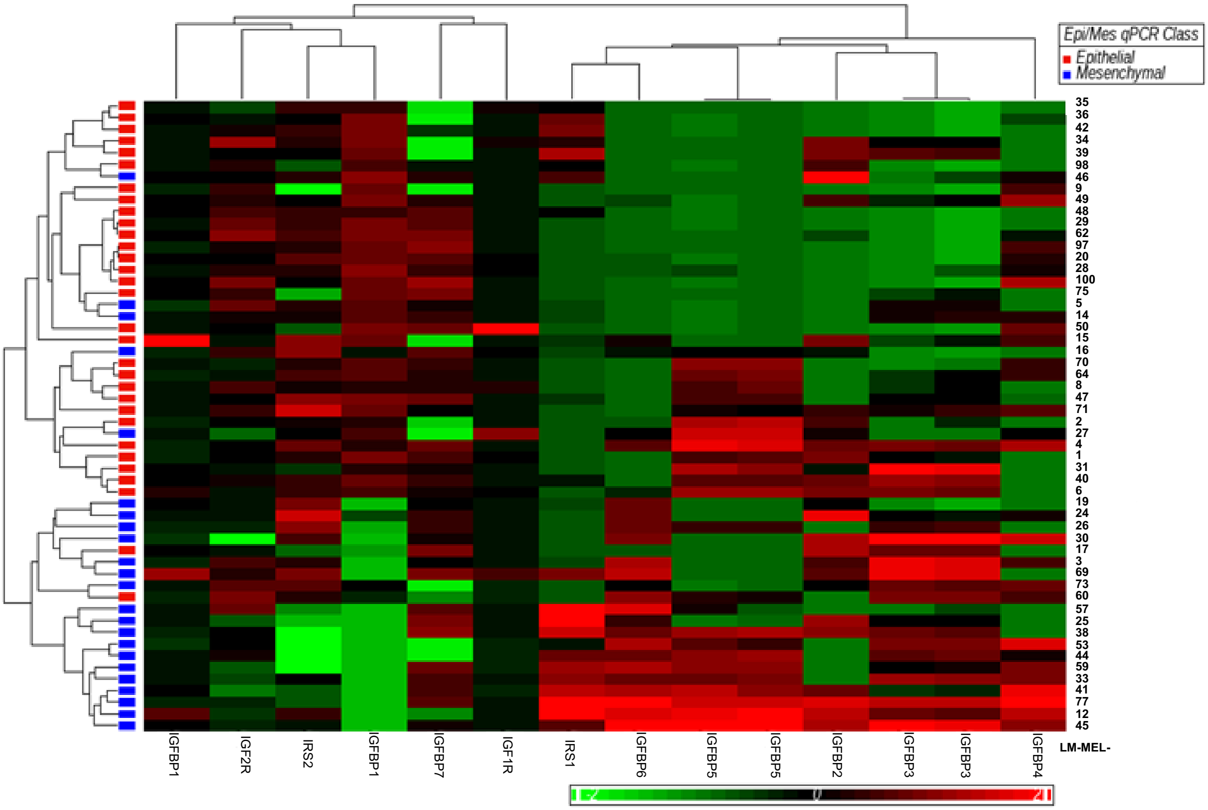

Supplementary Figure S3: Differential expression of IGF axis components in melanoma cells. Heat map shows hierarchical clustering of IGF axis components such as IGF receptors (*IGF-IR* and *IGF-IIR*), Insulin receptor substrates (*IRS1* and *IRS2*) and Insulin-like growth factor binding proteins (*IGFBP2–7*) across a panel of mesenchymal and epithelial melanoma cell lines.

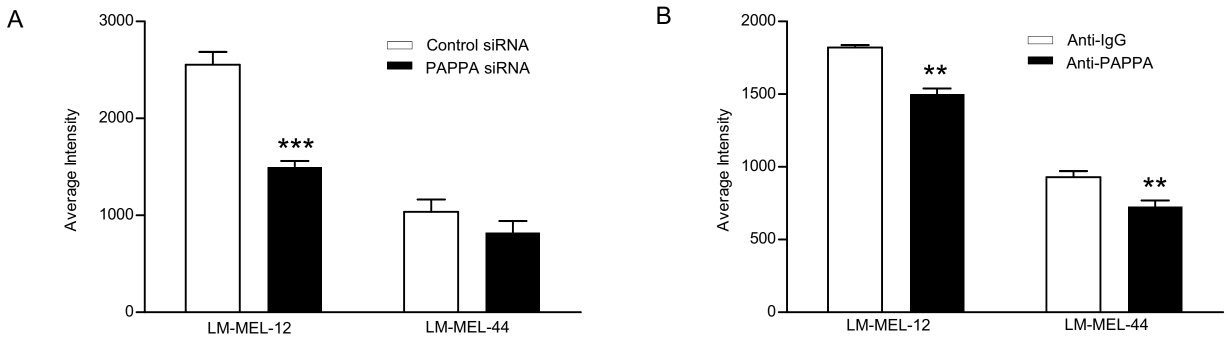

Supplementary Figure S4: Loss of PAPPA reduces invasive potential of melanoma cells *in vitro*. A. Melanoma cells were transfected with *PAPPA* siRNAs as previously described, the graphs show the total number of invading cells measured as intensity of invasive cells after crystal violet staining. **B.** Invasive ability of melanoma cells after treatment with control anti-IgG or anti-PAPPA antibody were quantified as above. Values are mean +/– SEM of four independent experiments in triplicate (**$p < 0.005$, ***$p < 0.0005$).

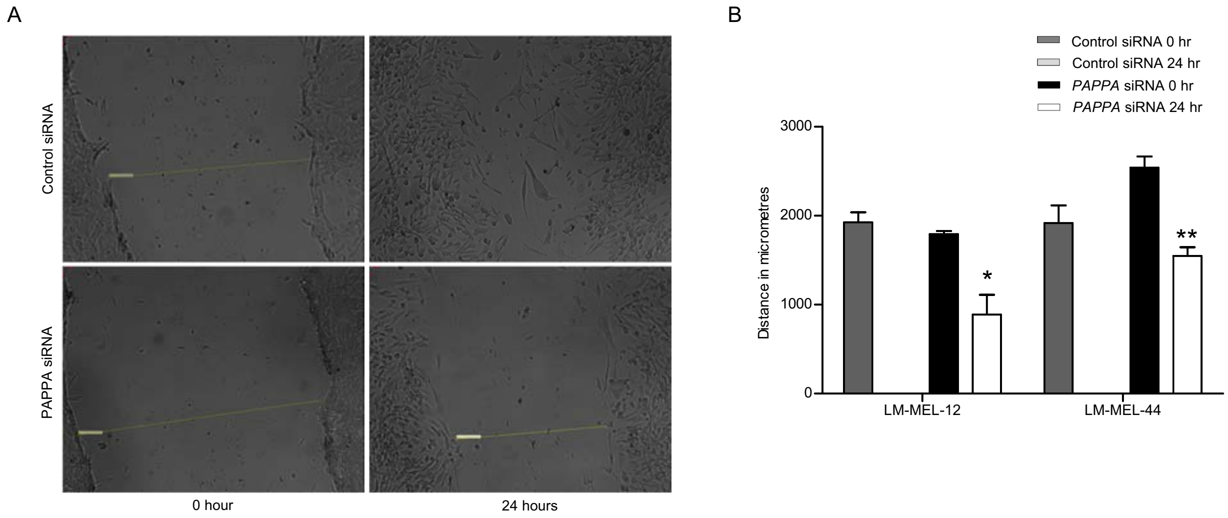

Supplementary Figure S5: Downregulation of PAPPA impairs wound healing ability in melanoma cells. **A.** LM-MEL-12 melanoma cells treated with control or PAPPA siRNA were subjected to wound healing assays. Representative images shown were captured at 0 and 24 hours (scale bar = 100 µm). **B.** Gap width was determined in LM-MEL-12 and LM-MEL-44 at indicated time points. Values are mean +/– SEM of three independent experiments in triplicate (*$p < 0.05$ **$p < 0.005$).

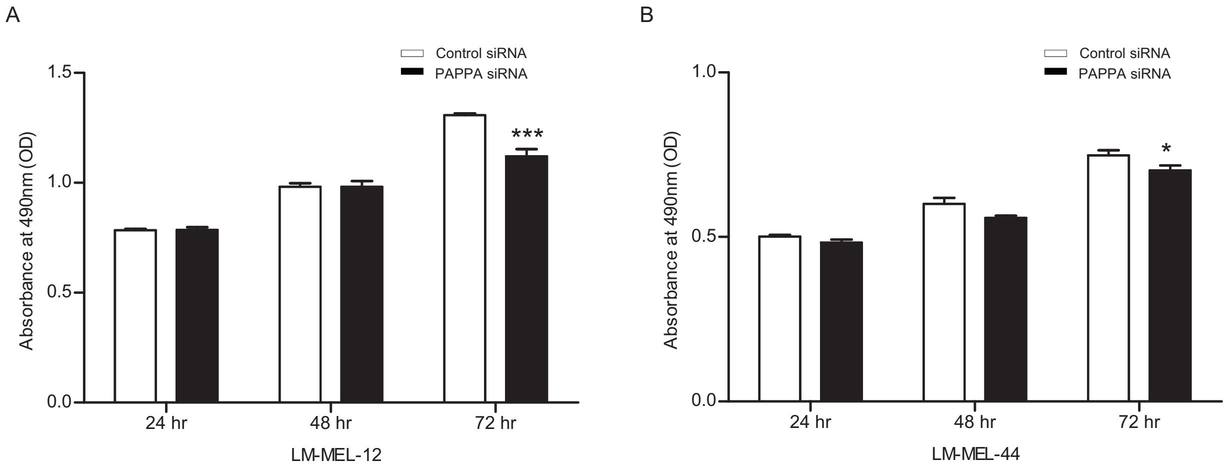

Supplementary Figure S6: Silencing of PAPPA attenuates proliferation ability in melanoma cells. A. LM-MEL-12 and **B.** LM-MEL-44 melanoma cells treated with control or PAPPA siRNA were subjected to MTS assay. Absorbance was assessed at indicated time points. Values are mean +/– SEM of three independent experiments in triplicate (*$p < 0.05$ ***$p < 0.0005$).

A

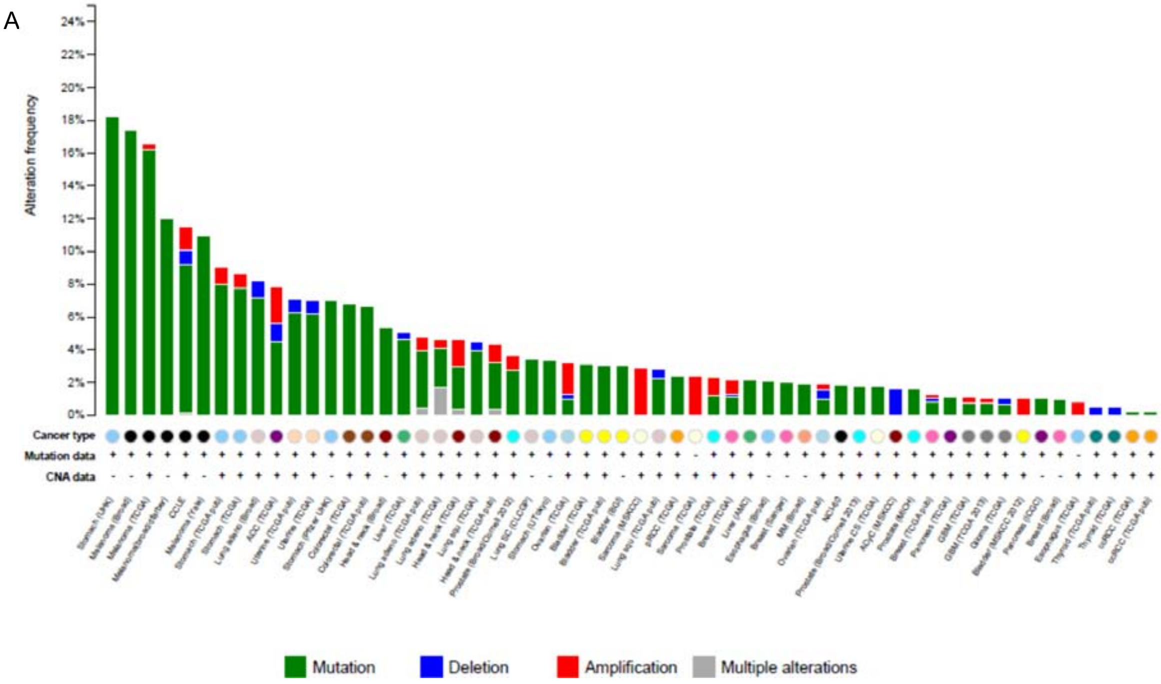

B

Gene	Co-occurrence (TCGA)
PAPPA & *IGF1*	**Odds Ratio: 3.79902 [tendency toward co-occurrence]**<br>95% Confidence Interval: 1.195764 - 12.069728<br>p-value: 0.03131 [Fisher's Exact Test]
PAPPA & *NCAD (CDH2)*	**Odds Ratio: 2.663043 [tendency toward co-occurrence]**<br>95% Confidence Interval: 1.19598 - 5.929699<br>p-value: 0.018298 [Fisher's Exact Test]

Supplementary Figure S7: Genomic alterations of PAPPA across a panel of human tumors. A. The results are displayed as histogram of alteration frequencies of *PAPPA* gene across a panel of human cancers queried in TCGA dataset. **B.** *PAPPA* shows a tendency towards mutual co-occurrence with *IGF1* and *N-cadherin* in melanomas tumors.
